# A Diversion

**Published:** 1920-08-07

**Authors:** 


### THE PUBLIC WELL-BEING.

### A Diversion.

### MR. HILAIRE BELLOC AND EUGENICS.

A number of famous and a vast number of very ordinary literary people seem inclined to act upon a topsy-turvy principle that literature is the training for life rather than life the training for literature. Write a few novels, and then one is able to emerge fully-equipped to teach statesmen, generals, reformers, and all other sections of people the subtleties of their several jobs. There were lots of them during the war who knew all about everything. Some, like Mr. Wells, Mr. Bennett, Mr. Galsworthy, and Mr. Belloc did know a good deal, but even Mr. Belloc's intricate arithmetical exercises —to say the least—did not always work out to the right answers. Still, he was entitled to bring his vast knowledge to bear upon the problems, just as a man of vision like Mr. Wells can always bring illumination to any subject upon which he fixes his amazingly vital intellect. Mr. Masefield lived in the war and made literature of it in his little masterpiece "Gallipoli," and there are obviously many examples of the uses made in the real functions of literature.

"In peace, we are in danger of being still further lessoned" (as Mr. Joseph Chamberlain once said) by big and small literary fry. They write books, and review those of others. They write plays, and criticise the plays of other dramatists. They expound their own ideas about the developments of art and poetry. But that is not all. They set out to run India (Mrs. Besant); they know what Ireland should have (Mr. Robert Lynd and a host of literary Irishmen); they are authorities on orthodoxy and the opposite (Mr. Chesterton and Mr. Shaw); they all have definitely didactic views on government in general, from Mr. Wells and Mr. Shaw down to the little garden suburb "highbrow" (and how he loves to be given that name). It is such a great temptation when one has a name and a market. Yet there are plenty of wise people who may even have greater knowledge and saner appreciation of the very things which these others regard as their own preserve.

#### Literature or Life.

At the same time, the fact that some of them have names gives their knowledge on other subjects an added power to influence. Take a case like Mr. Galsworthy. We speak of him because we have before us another letter of his putting in the right perspective some foolish pedant who, when Mr. Galsworthy wants to eliminate the ugly word "bastard" from legislation, reminds him that it is good old English. What Mr. Galsworthy objects to is that such a thing should be looked upon from the standpoint of the study of literature instead of from the standpoint of the study of life, and there is the whole matter. In the same paper as this letter from Mr. Galsworthy appeared was an attack on the Ministry of Health by Mr. Hilaire Belloc, who writes as a Catholic individualist much perturbed by some mysterious new devices—"most immoral and monstrous"—which the Ministry of Health is "certain to attempt." He cannot (he says) admit any interference with the liberty of marriage, nor any element at all of the horror known as eugenics. He cannot admit what is called sex-teaching, nor any of the whole series of propositions regarding sexual hygiene. Neither can he admit the claim of the State over the children as against the claim of the parents. So here is the doctrine of freedom of marriage, freedom to produce any kind of progeny, freedom to ignore all knowledge of sex, fierce opposition to any enlightenment, and denial of the duty of the State even to do what it can for the miserable children brought into the world by this detestable principle of *laisser-faire*. We are all for individual liberty, and against unnecessary interference by the State. But we profoundly oppose the doctrine that individual liberty means the liberty to wrong further generations by criminal neglect of essential knowledge. If Mr. Belloc's letter is correctly given it simply means that any diseased fool must—in the sacred name of liberty—be allowed to marry any other diseased fool, and have as many idiots for children as they may please. To use Mr. Belloc's own words, we say that that is a "most immoral and monstrous" plan. It is difficult to imagine that a man in his senses could advocate it, and we suspect that when Mr. Belloc speaks of "sex-teaching" and "sexual hygiene" he uses those terms with a meaning different from the one that seems apparent. Anyhow, eugenics does not mean the breeding of prize humans like prize cattle. It means, first of all, some careful precautions against spreading unutterable misery, and it is hard to imagine why that elementarily just principle should excite anyone's opposition, especially the opposition of any Christian church.

